# Dissecting the causal relationship between moderate to vigorous physical activity levels and cognitive performance: a bidirectional two-sample Mendelian randomization study

**DOI:** 10.3389/fpsyg.2024.1368241

**Published:** 2024-09-06

**Authors:** Qi Fang, Jinmin Zhang

**Affiliations:** ^1^Chengdu Sport University, Chengdu, Sichuan, China; ^2^School of Physical Education and Sport Science, Fujian Normal University, Fuzhou, China

**Keywords:** moderate to vigorous physical activity levels, cognitive performance, Mendelian randomization, health science, causal relationship

## Abstract

**Introduction:**

Recent studies increasingly suggest that moderate-to-vigorous physical activity (MVPA) impacts cognitive risk. However, the bidirectional nature of this relationship warrants further exploration. To address this, we employed a Mendelian randomization (MR) approach, analyzing two distinct samples.

**Methods:**

These analyses utilized published genome-wide association study (GWAS) summary statistics for MVPA (*n* = 377,234) and cognitive performance (*n* = 257,841). Our primary method was the inverse variance weighted (IVW) model with random effects, aiming to deduce potential causal links. Additionally, we employed supplementary methods, including MR Egger regression, Weighted median, Weighted mode, and Simple mode. For sensitivity analysis, tools like the MR Egger test, Cochran’s Q, MR PRESSO, and leave-one-out (LOO) were utilized.

**Results:**

Our findings indicate a decrease in cognitive risk with increased MVPA (Odds Ratio [OR] = 0.577, 95% Confidence Interval [CI]: 0.460–0.723, *p* = 1.930 × 10–6). Furthermore, enhanced cognitive levels corresponded to a reduced risk of inadequate MVPA (OR = 0.866, 95% CI: 0.839–0.895, *p* = 1.200 × 10–18).

**Discussion:**

In summary, our study demonstrates that MVPA lowers cognitive risk, while poor cognitive health may impede participation in MVPA. Overall, these findings provide valuable insights for developing personalized prevention and intervention strategies in health and sports sciences.

## 1 Introduction

Cognitive abilities encompass fluid ability (Gf) and crystallized ability (Gc). Gf includes skills like psychomotor speed, memory, and abstract reasoning, while Gc covers literacy, arithmetic, and professional knowledge and skills (Lövdén et al., 2020). These abilities are crucial predictors of educational and occupational success, socio-economic status, health, and longevity (Danesin et al., 2022). Cognitive decline can impair work capacity, social interactions, and daily living activities (Green, 1996). This is particularly concerning when cognitive deficits progress to clinically diagnosed dementia, such as Alzheimer’s disease or vascular dementia. Influential factors include age (Crivelli et al., 2022), educational level, socio-economic status (Ma et al., 2015), and physical fitness (Ritchie et al., 2016). The cognitive simulation hypothesis posits that physically demanding activities that also require significant cognitive engagement can notably enhance cognitive functions, including problem-solving, memory, and executive functions (Jackson et al., 2016).

Moderate-to-vigorous physical activity (MVPA) is defined as any exercise that yields ≥ 3.0 metabolic equivalents (METs). Regular participation in physical activity beneficially influences physical health, life quality, cognition, and mental health (Biddle et al., 2019). The World Health Organization’s recommendation on physical activity for health is to engage in at least 150 min of moderate-intensity or 75 min of high-intensity physical activity every week, or any equivalent combination of the two (WHO, 2010). Lack of physical activity (i.e., not engaging in ≥ 150 min/week of moderate to vigorous physical activity) is significantly associated with cognitive impairment (Falck et al., 2020). High-intensity and frequent physical activity may be the most effective exercise type for improving mild cognitive impairment and overall cognitive ability in adults (Lista and Sorrentino, 2010).

Currently, the results of epidemiological studies indicate that physical activity can enhance cognitive function in both young and older individuals (Lista and Sorrentino, 2010), attention processes (Kramer et al., 1999), and executive function (Chieffi et al., 2017). Furthermore, physical activity can prevent age-related cognitive decline (Niemann et al., 2014), reduce the risk of dementia (Colberg et al., 2008), and mitigate the extent of executive function deterioration (Hollamby et al., 2017). However, subsequent studies, such as those examining high-intensity interval training (HIIT) style vigorous physical activity (VPA), found no significant improvements in cognitive functions like executive function (EF), relational memory, and processing speed (Wassenaar et al., 2021). Even in studies focusing solely on VPA, no discernible benefits were observed (Haverkamp et al., 2020). This raises questions about the existence of a causal link between PA and cognitive performance. Additionally, in patients with cognitive impairments, such as those with schizophrenia, a common feature is an unhealthy lifestyle characterized by inadequate physical activity (Wichniak et al., 2011) and poor physical health (Mitchell and Malone, 2006). Thus, the potential bidirectional causal relationship between cognitive performance and physical activity remains unclear. Traditional observational research is limited by inherent flaws, including the inability to fully rule out reverse causality and confounding factors (Sekula et al., 2016). MR, in contrast, offers a robust approach to addressing these issues.

MR is an epidemiological technique that employs single nucleotide polymorphisms (SNPs) associated with an exposure (e.g., MVPA) as instrumental variables (IVs) to deduce potential causal relationships between the exposure and outcomes (Davey Smith and Hemani, 2014). Due to the principle of random assortment and combination in allele distribution, theoretically, the causal effect should be free from confounding factors and reverse causation (Davies et al., 2018). IVs can be derived from the published summary statistics of genome-wide association studies (GWAS), commonly used in two-sample MR studies (Porcu et al., 2019). This study aims to explore the bidirectional causal effects between MVPA and cognitive performance.

## 2 Materials and methods

### 2.1 Experimental design

Our MR analysis of the two samples utilized published genome-wide association study (GWAS) summary statistics and received ethical approval from the original study. The data sources for this study are detailed in Supplementary Table 1. We selected major SNPs associated with moderate to vigorous physical activity (MVPA) and cognitive performance as genetic instrumental variables (IVs) to conduct bidirectional two-sample MR. To minimize racial mismatches, our analysis was confined to participants of European descent.

Our selection of qualified genetic instruments involved a thorough quality control process. Initially, we included SNPs closely related to exposure factors at a significance threshold of 10–8. We then employed an R2 < 0.001 and a window size of 10,000 kb for clustering. In cases where the target SNP was unavailable in the result dataset, a proxy SNP with an R2 > 0.8 was used as a substitute. Furthermore, the minor allele frequency of the SNP in the GWAS findings was set at a default value of 0.3. Finally, we aligned exposure and outcome data to exclude palindromic SNPs and ensure that the effective allele was consistent across both datasets (Hartwig et al., 2016). The statistical power of the SNP is calculated using an online tool, and the website is https://shiny.cnsgenomics.com/mRnd/ (Brion et al., 2013). Therefore, our research design is outlined as follows: (1) Identification of exposure-related SNPs under a genome-wide significance threshold; (2) Independence of the SNP from potential confounding factors; (3) Influence of the SNP on the outcome exclusively through exposure.

### 2.2 Data sources

For MVPA, we used publicly available GWAS data (Guthold et al., 2018) from a cohort of 377,234 participants. Using genetic variables related to MVPA as tools, 19 SNPs were identified as significantly associated with MVPA (*P* < 5 × 10–8) and met the IV criteria (Supplementary Table 2). In our study, F statistic > 29.984 for each SNP (Supplementary Table 3).

The cognitive performance data was sourced from a GWAS (Lee et al., 2018) involving 257,841 participants. Here, 147 SNPs were identified as significantly related to cognitive performance (*P* < 5 × 10–8), with 139 of these SNPs meeting the IV criteria (Supplementary Table 4). And F statistic > 29.793 for each SNP (Supplementary Table 5).

### 2.3 Statistical analysis

In this study, all analyses were conducted using the TwoSampleMR package (version 0.5.8) and MR-PRESSO (version 1.0) in R software (version 4.3.2). The original data and code files are stored in the Open Science Framework.^1^ Our primary method for MR was the IVW regression. This approach is widely used for the main analyses in various studies. The IVW model synthesizes the Wald ratios of each single nucleotide polymorphism (SNP) to yield a combined causal estimate (Chen et al., 2022). The IVW model’s results are presented as odds ratios (ORs) with corresponding 95% Confidence Intervals (CIs). Additionally, we employed complementary methods, including MR Egger regression, the weighted median, the simple model, and the weighted model, to assess the causal impact of exposure on outcomes. The MR Egger approach relaxes the “no pleiotropy” assumption and allows for a non-zero intercept (Hemani et al., 2018a). The weighted median method considers the median effect of all available SNPs, assuming that at least half of the SNPs are valid instruments.

### 2.4 Sensitivity analysis

We performed a series of sensitivity analyses to evaluate the robustness and validity of our causal estimates. Cochran’s Q statistic was used to assess the heterogeneity among individual effect estimates for each genetic variation. A non-significant Q value may indicate the absence of pleiotropy to some extent (Hemani et al., 2018b). The MR-PRESSO test detects pleiotropy and reassesses the association via IVW after excluding pleiotropic SNPs (Verbanck et al., 2018). The MR Egger intercept test evaluates the directional pleiotropy, a key consideration in IVW estimates (Bowden et al., 2015). Funnel plots were also employed to assess directional horizontal pleiotropy, with a symmetrical distribution suggesting the absence of directional pleiotropy. Lastly, the leave-one-out (LOO) method was used to determine if the aggregated estimates are disproportionately influenced by any single genetic variation (Liu et al., 2021).

### 2.5 Explanatory measures

Cognitive ability refers to the brain’s capacity to process, store, and retrieve information. As a comprehensive cognitive measure, it encompasses both general and specific abilities in various domains, such as working memory, attention, visual and spatial processing, information processing speed, and executive function (Zimmer et al., 2016). Cognitive performance represents the abilities and behavioral characteristics exhibited by individuals during cognitive processes, such as language, perception, and arithmetic tests. In this study, the measurement criteria for cognitive performance were the respondents’ scores on a language cognition test, combined with scores from a cognitive test in the Wisconsin Longitudinal Study (WLS). Cognitive risk refers to the likelihood of an individual experiencing a decline, impairment, or disorder in cognitive ability and performance. MVPA level denotes moderate to high levels of VPA (75–300 min/week) or MPA (150–600 min/week) or any equivalent combination of the two (Lee et al., 2022).

## 3 Results

### 3.1 The causal effect of MVPA on cognitive performance

**FIGURE 1:**
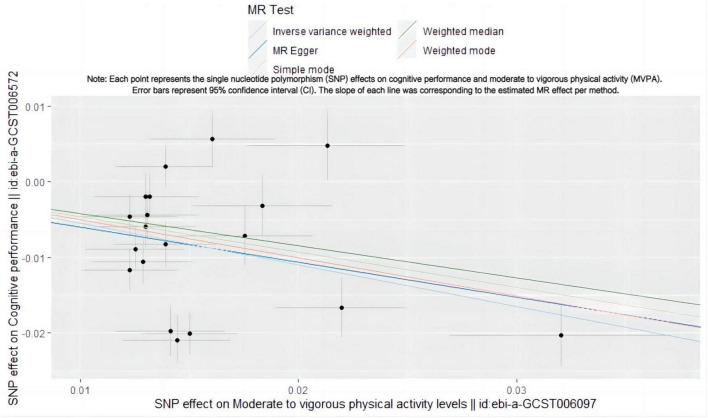
Scatter plot illustrating the SNP effect of MVPA on cognitive performance. Each point represents the single nucleotide polymorphism (SNP) effects on cognitive performance and moderate to vigorous physical activity (MVPA). Error bars represent 95% confidence interval (CI). The slope of each line was corresponding to the estimated MR effect per method.

The MR analysis revealed that MVPA acts as a protective factor against cognitive risk (Figure 1). A logarithmic odds ratio (OR) increase in MVPA correlated with a 42.3% decrease in cognitive risk (IVW OR = 0.577, 95% CI: 0.460–0.723, *p* = 1.930 × 10–6) (Table 1 and Supplementary Figure 1), a statistically significant difference.

**TABLE 1:** Mendelian randomization results of MVPA on cognitive performance.

Method	SNPs	Or	or_lci95	or_uci95	*P*	Q statistic *P*-value**	MR-Egger pleiotropy test *P*-value	F-statistical
MR Egger	19	0.629462971	0.254570637	1.556438858	0.330309809			
Weighted median	19	0.654153	0.549593616	0.778604655	1.78546E-06			
Inverse variance weighted	19	0.576571056	0.459614748	0.723288763	1.92969E-06	5.52985E-14	0.846433645	3811.44
Simple mode	19	0.627169785	0.434202836	0.90589445	0.022927511			
Weighted mode	19	0.604286764	0.437512315	0.834633633	0.006786851			

When Cochran’s Q statistics *P* ≤ 0.05, random-effect modal IVW MR analysis was used. IVW, inverse variance weighted method; SNP, single nucleotide polymorphism; OR, odds ratio; CI, confidence interval.

**Statistically significant.

Cochran’s Q statistics were employed to evaluate heterogeneity, which could be attributed to variations in experimental platforms. However, as our IVW model is a random-effects model, this heterogeneity does not compromise our results. The MR Egger test assessed directional pleiotropy (*p* = 0.846), finding no significant directional pleiotropy in all results. The funnel plot also indicated a low risk of directional pleiotropy in our IVW estimates (Supplementary Figure 2). The stability of the results was confirmed through single SNP exclusion analysis (Supplementary Figure 3).

### 3.2 Causality of cognitive performance on MVPA

**FIGURE 2:**
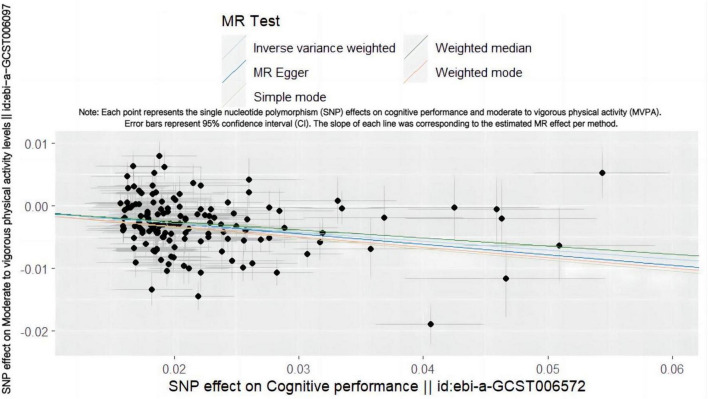
Scatter plot showing the SNP effect of cognitive performance on MVPA. Each point represents the single nucleotide polymorphism (SNP) effects on cognitive performance and moderate to vigorous physical activity (MVPA). Error bars represent 95% confidence interval (CI). The slope of each line was corresponding to the estimated MR effect per method.

Conversely, when cognitive genetic predisposition was considered as the exposure, MR analysis showed a decrease in MVPA deficiency risk with improved cognitive performance (Figure 2). This suggests a protective causal relationship between cognitive performance and MVPA (IVW OR = 0.866, 95% CI: 0.839–0.895, *p* = 1.200 × 10–18) (Table 2 and Supplementary Figure 4), which was statistically significant.

**TABLE 2:** Mendelian randomization results of cognitive performance on MVPA.

Method	SNPs	Or	or_lci95	or_uci95	*P*	Q statistic *P*-value**	MR-Egger pleiotropy test *P*-value	F-statistical
MR Egger	139	0.846149349	0.735624541	0.973280091	0.020769038			
Weighted median	139	0.878192805	0.851094832	0.906153548	4.56396E-16			
Inverse variance weighted	139	0.866448892	0.839266264	0.894511927	1.19983E-18	1.3276E-22	0.733644572	2605.45
Simple mode	139	0.839176399	0.751766257	0.936749984	0.002172954			
Weighted mode	139	0.846616721	0.768373235	0.932827745	0.000989994			

When Cochran’s Q statistics *P* ≤ 0.05, random-effect modal IVW MR analysis was used. IVW, inverse variance weighted method; SNP, single nucleotide polymorphism; OR, odds ratio; CI, confidence interval.

**Statistically significant.

Cochran’s Q statistics assessed heterogeneity. MR-PRESSO identified 8 pleiotropic SNPs out of 147, yet reanalysis after outlier removal did not materially alter results. All MR Egger intercept tests indicated no horizontal pleiotropy (*p* > 0.05, intercept close to zero). The funnel plot was approximately symmetrical on both sides, suggesting no evident bias (Supplementary Figure 5). Leave-one-out (LOO) analysis confirmed that no single SNP significantly altered the causal effect of MVPA on cognitive performance (Supplementary Figure 6).

## 4 Discussion

In our MR study, a significant potential causal relationship between MVPA and cognitive performance was identified. We observed that MVPA has a protective effect. Conversely, the MR results suggest a causal link between cognitive impairment and insufficient MVPA. The robustness of these results is supported by comprehensive sensitivity analyses and multiple validity evaluations.

Our research aligns with previous studies, affirming that adequate MVPA significantly reduces cognitive risk. This protective causal effect can be attributed to MVPA’s association with enhanced cognitive functions, as supported by Savilla et al. (2008). One plausible explanation is that improved cognitive performance correlates with increased volume in critical brain regions like the prefrontal cortex (involved in working memory), the temporal cortex (engaged in perception and semantic representation), and the medial orbitofrontal cortex (Cheng et al., 2021). Higher levels of MVPA are linked to increased cortical thickness in the left superior frontal gyrus and the right temporal pole. Thinning of the frontal and temporal cortices is associated with aging and dementia (Salat et al., 2004); hence, higher MVPA is crucial for maintaining the cortical thickness in areas vulnerable to cognitive decline (Cheng et al., 2021). Additionally, MVPA correlates with greater total gray matter volume, enhancing insulin sensitivity and cognitive function through exercise training. This suggests that substantial MVPA can protect brain health (Falck et al., 2020). Moreover, MVPA improves aerobic fitness and cardiovascular health, which in turn benefits higher-order cognitive functions like executive function. This improvement may be mediated by neurophysiological changes in prefrontal cortex oxygenation related to aerobic fitness levels (James et al., 2023). In aging populations, continual MVPA can mitigate brain aging changes and stimulate repair processes, thereby reducing cognitive decline risks (Köhncke et al., 2018; Dominguez et al., 2021). For individuals with lower cognitive abilities, MVPA improves cognitive performance, including executive function, language, working memory, and processing speed, as observed in schizophrenia patients (Kimhy et al., 2014). A potential mechanism is the upregulation of brain-derived neurotrophic factor (BDNF) during exercise, which preliminary evidence suggests may mediate cognitive improvements in schizophrenia (Firth et al., 2017). Additionally, MVPA is a beneficial intervention for Parkinson’s patients, potentially due to its role in inducing neuroprotection, preventing cell death, and regulating mitochondrial function and neuroinflammation by promoting autophagy and neuronal regeneration (Liao et al., 2022). Through two-way MR, we discovered that individuals with lower cognitive levels may be less inclined to engage in MVPA. This observation aligns with existing research suggesting that reduced cognitive capacity can hinder participation in MVPA. For instance, patients with mental illnesses often show poor adherence to exercise interventions, tending toward lower levels of physical activity and reduced engagement in intense exercise, while sedentary behaviors significantly increase. This could be attributed to factors such as lack of motivation, cognitive decline, psycho-pathological symptoms, and medication side effects, which make achieving optimal physical activity levels challenging (Vila-Barrios et al., 2023). Additionally, early prodromal symptoms in Parkinson’s disease patients, including hypoesthesia, decreased perception, and sleep disorders, which may manifest up to 20 years before diagnosis, could diminish their inclination toward physical activity. This decrease in activity may be linked to early dopamine loss, resulting in reduced participation in physical activities (Baumeister et al., 2021).

Furthermore, in this study, we can extend the discussion to the relationship between exercise reserve and cognition. Exercise reserve refers to an individual’s abilities or resources in physical activity or exercise that help maintain or improve physical activity levels when needed, such as cardiorespiratory function, muscle strength, flexibility, and balance. Physical activity is defined as any bodily movement produced by skeletal muscles that results in energy expenditure, while exercise is a subset of planned, structured, and repetitive physical activity with the ultimate or intermediate objective of improving or maintaining physical fitness (Caspersen et al., 1985). The role of exercise reserve includes improving physical health, enhancing cognitive function, delaying the aging process, and ultimately improving quality of life. Specifically, there is a close relationship between exercise reserve and physical activity. On one hand, physical activity is the primary means to enhance and maintain exercise reserve. For example, physical activities involving large muscle groups are associated with arm and lower limb activities, which can increase muscle strength, balance, and mobility (Weinstein et al., 2023). On the other hand, a good exercise reserve helps individuals reduce exercise fatigue and lower the risk of injury during physical activity. Exercise fatigue refers to the inability to maintain the required force during sustained muscle contraction (Holtzer et al., 2011). Consistent with this study, there is also a close relationship between exercise reserve and cognition. Firstly, research findings have shown that increasing total daily activity levels and exercise capacity can independently improve cognitive abilities and reduce dementia (Buchman et al., 2019). For instance, a recent report indicated that combining aerobic and resistance training improved overall cognition and subdomains such as attention, visuospatial function, and executive function (Marzolini et al., 2013). Physical activity can reduce the risk of cognitive impairment in independently living older adults, primarily vascular dementia (Verdelho et al., 2012). Conversely, high levels of cognitive function can enhance people’s mastery and understanding of exercise, especially skilled motor performance and fine motor performance (e.g., handwriting and manual dexterity tasks) (Liou et al., 2020). In contrast, a lack of exercise and prolonged sedentary behavior may lead to cognitive decline and even increase the risk of chronic diseases. For example, individuals with autism often exhibit decreased levels of physical activity. Fewer opportunities for physical activity are more likely to affect their verbal communication, social adaptation, and behavioral difficulties (Pan and Frey, 2006) and lead to some chronic diseases, such as obesity, which is common in individuals with autism (Tyler et al., 2011).

From an individual perspective, we strongly advocate for all adults to engage in at least 150 min per week of MVPA to maintain brain health (Falck et al., 2017) and lessen the risk of age-related cognitive disorders. Cognition-enhancing MVPAs include activities such as running, jumping, rope skipping, active cycling, ice hockey, playing ball games, karate, table tennis, mixed martial arts, and team sports (Peiris et al., 2022). Healthy older adults can derive cognitive benefits from increasing their MVPA, even at moderate intensities (e.g., approximately 110 steps/minute). For individuals with cognitive disorders, physical activity interventions can aid in improving short-term memory, working memory, and processing speed (Chen et al., 2016). On a national level, we recommend the implementation of effective policies to encourage non-motorized commuting, such as walking and cycling, by enhancing infrastructure and road safety. Creating more opportunities for sports activities in public open spaces, parks, workplaces, and local communities can motivate residents to participate in active recreation and sports activities in their free time.

## 5 Advantages and limitations

This study extracts genetic instruments from the largest and most recent GWAS of MVPA and cognitive performance. Employing a univariate MR design, a series of sensitivity analyses are conducted to control for pleiotropy bias and validate the robustness of the MR results. Our research using genetic instruments reveals a potential bidirectional causal relationship between MVPA and cognitive performance.

However, it is important to acknowledge certain limitations. The GWAS summary data utilized in this study encompasses only individuals of European descent. Consequently, the applicability of our findings to other populations requires further validation. Additionally, due to the limited information in the aggregated data, we are unable to ascertain if gender influences our results.

## 6 Conclusion

We discovered that MVPA can mitigate cognitive risk, yet a low cognitive level also contributes to inadequate MVPA. This reciprocal causal relationship between MVPA deficiency and cognitive risk may indicate a detrimental cycle, posing an additional risk factor for increased cognitive impairment. These findings offer valuable insights for the formulation of policies aimed at preventing and intervening in cognitive risks. Our research provides evidence to assist individuals in selecting the appropriate quantity and intensity of physical activity throughout their lives to preserve their overall health.

## Data Availability

The original contributions presented in this study are included in the article/Supplementary material, further inquiries can be directed to the corresponding author.

## Footnotes

1
https://osf.io/bhp4c/?view_only=75466f8f48fe4d0494cedd31a18c0422

## References

[B1] Baumeister S. Meisinger C. Leitzmann M. Teumer A. Bahls M. Karch A. (2021). Physical activity and Parkinson’s disease: A two-sample Mendelian randomisation study. *J. Neurol. Neurosurg. Psychiatry* 92 334–335. 10.1136/jnnp-2020-324515 33093192

[B2] Biddle S. J. H. Ciaccioni S. Thomas G. Vergeer I. (2019). Physical activity and mental health in children and adolescents: An updated review of reviews and an analysis of causality. *Psychol. Sport Exerc.* 42 146–155. 10.1016/j.psychsport.2018.08.011

[B3] Bowden J. Davey Smith G. Burgess S. (2015). Mendelian randomization with invalid instruments: Effect estimation and bias detection through Egger regression. *Int. J. Epidemiol.* 44 512–525. 10.1093/ije/dyv080 26050253 PMC4469799

[B4] Brion M. J. Shakhbazov K. Visscher P. M. (2013). Calculating statistical power in Mendelian randomization studies. *Int. J. Epidemiol.* 42 1497–1501. 10.1093/ije/dyt179 24159078 PMC3807619

[B5] Buchman A. S. Yu L. Wilson R. S. Lim A. Dawe R. J. Gaiteri C. (2019). Physical activity, common brain pathologies, and cognition in community-dwelling older adults. *Neurology* 92 e811–e822. 10.1212/wnl.0000000000006954 30651386 PMC6396972

[B6] Caspersen C. J. Powell K. E. Christenson G. M. (1985). Physical activity, exercise, and physical fitness: Definitions and distinctions for health-related research. *Public Health Rep.* 100 126–131. 3920711 PMC1424733

[B7] Chen L. J. Steptoe A. Chung M. S. Ku P. W. (2016). Association between actigraphy-derived physical activity and cognitive performance in patients with schizophrenia. *Psychol. Med.* 46 2375–2384. 10.1017/s0033291716000921 27283122

[B8] Chen X. Hong X. Gao W. Luo S. Cai J. Liu G. (2022). Causal relationship between physical activity, leisure sedentary behaviors and COVID-19 risk: A Mendelian randomization study. *J. Transl. Med.* 20:216. 10.1186/s12967-022-03407-6 35562752 PMC9100292

[B9] Cheng W. Rolls E. Gong W. Du J. Zhang J. Zhang X. Y. (2021). Sleep duration, brain structure, and psychiatric and cognitive problems in children. *Mol. Psychiatry* 26 3992–4003. 10.1038/s41380-020-0663-2 32015467 PMC8855973

[B10] Chieffi S. Messina G. Villano I. Messina A. Valenzano A. Moscatelli F. (2017). Neuroprotective effects of physical activity: Evidence from human and animal studies. *Front. Neurol.* 8:188. 10.3389/fneur.2017.00188 28588546 PMC5439530

[B11] Colberg S. R. Somma C. T. Sechrist S. R. (2008). Physical activity participation may offset some of the negative impact of diabetes on cognitive function. *J. Am. Med. Dir. Assoc.* 9 434–438. 10.1016/j.jamda.2008.03.014 18585646

[B12] Crivelli L. Palmer K. Calandri I. Guekht A. Beghi E. Carroll W. (2022). Changes in cognitive functioning after COVID-19: A systematic review and meta-analysis. *Alzheimers Dement.* 18 1047–1066. 10.1002/alz.12644 35297561 PMC9073922

[B13] Danesin L. Giustiniani A. Arcara G. Burgio F. (2022). Financial decision-making in neurological patients. *Brain Sci.* 12:529. 10.3390/brainsci12050529 35624916 PMC9139159

[B14] Davey Smith G. Hemani G. (2014). Mendelian randomization: Genetic anchors for causal inference in epidemiological studies. *Hum. Mol. Genet.* 23 R89–R98. 10.1093/hmg/ddu328 25064373 PMC4170722

[B15] Davies N. M. Holmes M. V. Davey Smith G. (2018). Reading Mendelian randomisation studies: A guide, glossary, and checklist for clinicians. *BMJ* 362:k601. 10.1136/bmj.k601 30002074 PMC6041728

[B16] Dominguez L. J. Veronese N. Vernuccio L. Catanese G. Inzerillo F. Salemi G. (2021). Nutrition, physical activity, and other lifestyle factors in the prevention of cognitive decline and dementia. *Nutrients* 13:4080. 10.3390/nu13114080 34836334 PMC8624903

[B17] Falck R. S. Davis J. C. Liu-Ambrose T. (2017). What is the association between sedentary behaviour and cognitive function? A systematic review. *Br. J. Sports Med.* 51 800–811. 10.1136/bjsports-2015-095551 27153869

[B18] Falck R. S. Hsu C. L. Best J. R. Li L. C. Egbert A. R. Liu-Ambrose T. (2020). Not just for joints: The associations of moderate-to-vigorous physical activity and sedentary behavior with brain cortical thickness. *Med. Sci. Sports Exerc.* 52 2217–2223. 10.1249/mss.0000000000002374 32936595

[B19] Firth J. Stubbs B. Rosenbaum S. Vancampfort D. Malchow B. Schuch F. (2017). Aerobic exercise improves cognitive functioning in people with schizophrenia: A systematic review and meta-analysis. *Schizophr. Bull.* 43 546–556. 10.1093/schbul/sbw115 27521348 PMC5464163

[B20] Green M. F. (1996). What are the functional consequences of neurocognitive deficits in schizophrenia? *Am. J. Psychiatry* 153 321–330. 10.1176/ajp.153.3.321 8610818

[B21] Guthold R. Stevens G. A. Riley L. M. Bull F. C. (2018). Worldwide trends in insufficient physical activity from 2001 to 2016: A pooled analysis of 358 population-based surveys with 19 million participants. *Lancet Glob. Health* 6 e1077–e1086. 10.1016/s2214-109x(18)30357-7 30193830

[B22] Hartwig F. P. Davies N. M. Hemani G. Davey Smith G. (2016). Two-sample Mendelian randomization: Avoiding the downsides of a powerful, widely applicable but potentially fallible technique. *Int. J. Epidemiol.* 45 1717–1726. 10.1093/ije/dyx028 28338968 PMC5722032

[B23] Haverkamp B. F. Wiersma R. Vertessen K. van Ewijk H. Oosterlaan J. Hartman E. (2020). Effects of physical activity interventions on cognitive outcomes and academic performance in adolescents and young adults: A meta-analysis. *J. Sports Sci.* 38 2637–2660. 10.1080/02640414.2020.1794763 32783695

[B24] Hemani G. Bowden J. Davey Smith G. (2018a). Evaluating the potential role of pleiotropy in Mendelian randomization studies. *Hum. Mol. Genet.* 27 R195–R208. 10.1093/hmg/ddy163 29771313 PMC6061876

[B25] Hemani G. Zheng J. Elsworth B. Wade K. H. Haberland V. Baird D. (2018b). The MR-Base platform supports systematic causal inference across the human phenome. *Elife* 7:e34408. 10.7554/eLife.34408 29846171 PMC5976434

[B26] Hollamby A. Davelaar E. J. Cadar D. (2017). Increased physical fitness is associated with higher executive functioning in people with dementia. *Front. Public Health* 5:346. 10.3389/fpubh.2017.00346 29312919 PMC5742628

[B27] Holtzer R. Shuman M. Mahoney J. R. Lipton R. Verghese J. (2011). Cognitive fatigue defined in the context of attention networks. *Neuropsychol. Dev. Cogn. B Aging Neuropsychol. Cogn.* 18 108–128. 10.1080/13825585.2010.517826 21128132 PMC3058923

[B28] Jackson W. M. Davis N. Sands S. A. Whittington R. A. Sun L. S. (2016). Physical activity and cognitive development: A meta-analysis. *J. Neurosurg. Anesthesiol.* 28 373–380. 10.1097/ana.0000000000000349 27768674

[B29] James J. Pringle A. Mourton S. Roscoe C. M. P. (2023). The effects of physical activity on academic performance in school-aged children: A systematic review. *Children (Basel)* 10:1019. 10.3390/children10061019 37371251 PMC10297707

[B30] Kimhy D. Vakhrusheva J. Bartels M. N. Armstrong H. F. Ballon J. S. Khan S. (2014). Aerobic fitness and body mass index in individuals with schizophrenia: Implications for neurocognition and daily functioning. *Psychiatry Res.* 220 784–791. 10.1016/j.psychres.2014.08.052 25219618 PMC4258141

[B31] Köhncke Y. Papenberg G. Jonasson L. Karalija N. Wåhlin A. Salami A. (2018). Self-rated intensity of habitual physical activities is positively associated with dopamine D(2/3) receptor availability and cognition. *Neuroimage* 181 605–616. 10.1016/j.neuroimage.2018.07.036 30041059

[B32] Kramer A. F. Hahn S. Cohen N. J. Banich M. T. McAuley E. Harrison C. R. (1999). Ageing, fitness and neurocognitive function. *Nature* 400 418–419. 10.1038/22682 10440369

[B33] Lee D. H. Rezende L. F. M. Joh H. K. Keum N. Ferrari G. Rey-Lopez J. P. (2022). Long-term leisure-time physical activity intensity and all-cause and cause-specific mortality: A prospective cohort of US adults. *Circulation* 146 523–534. 10.1161/circulationaha.121.058162 35876019 PMC9378548

[B34] Lee J. J. Wedow R. Okbay A. Kong E. Maghzian O. Zacher M. (2018). Gene discovery and polygenic prediction from a genome-wide association study of educational attainment in 1.1 million individuals. *Nat. Genet.* 50 1112–1121. 10.1038/s41588-018-0147-3 30038396 PMC6393768

[B35] Liao Q. He J. Huang K. (2022). Physical activities and risk of neurodegenerative diseases: A two-sample Mendelian randomization study. *Front. Aging Neurosci.* 14:991140. 10.3389/fnagi.2022.991140 36212040 PMC9541335

[B36] Liou W. C. Chan L. Hong C. T. Chi W. C. Yen C. F. Liao H. F. (2020). Hand fine motor skill disability correlates with dementia severity. *Arch. Gerontol. Geriatr.* 90:104168. 10.1016/j.archger.2020.104168 32650157

[B37] Lista I. Sorrentino G. (2010). Biological mechanisms of physical activity in preventing cognitive decline. *Cell Mol. Neurobiol.* 30 493–503. 10.1007/s10571-009-9488-x 20041290 PMC11498799

[B38] Liu W. Zhang L. Li S. Liu C. Tong Y. Fang H. (2021). A Mendelian randomization study of plasma homocysteine levels and cerebrovascular and neurodegenerative diseases. *Front. Genet.* 12:653032. 10.3389/fgene.2021.653032 33868384 PMC8047106

[B39] Lövdén M. Fratiglioni L. Glymour M. M. Lindenberger U. Tucker-Drob E. M. (2020). Education and cognitive functioning across the life span. *Psychol. Sci. Public Interest.* 21 6–41. 10.1177/1529100620920576 32772803 PMC7425377

[B40] Ma J. K. Le Mare L. Gurd B. J. (2015). Four minutes of in-class high-intensity interval activity improves selective attention in 9- to 11-year olds. *Appl. Physiol. Nutr. Metab.* 40 238–244. 10.1139/apnm-2014-0309 25675352

[B41] Marzolini S. Oh P. McIlroy W. Brooks D. (2013). The effects of an aerobic and resistance exercise training program on cognition following stroke. *Neurorehabil. Neural Repair* 27 392–402. 10.1177/1545968312465192 23161865

[B42] Mitchell A. J. Malone D. (2006). Physical health and schizophrenia. *Curr. Opin. Psychiatry* 19 432–437. 10.1097/01.yco.0000228767.71473.9e 16721177

[B43] Niemann C. Godde B. Staudinger U. M. Voelcker-Rehage C. (2014). Exercise-induced changes in basal ganglia volume and cognition in older adults. *Neuroscience* 281 147–163. 10.1016/j.neuroscience.2014.09.033 25255932

[B44] Pan C. Y. Frey G. C. (2006). Physical activity patterns in youth with autism spectrum disorders. *J. Autism Dev. Disord.* 36 597–606. 10.1007/s10803-006-0101-6 16652237

[B45] Peiris D. Duan Y. Vandelanotte C. Liang W. Yang M. Baker J. S. (2022). Effects of in-classroom physical activity breaks on children’s academic performance, cognition, health behaviours and health outcomes: A systematic review and meta-analysis of randomised controlled trials. *Int. J. Environ. Res. Public Health* 19:9479. 10.3390/ijerph19159479 35954831 PMC9368257

[B46] Porcu E. Rüeger S. Lepik K. Santoni F. A. Reymond A. Kutalik Z. (2019). Mendelian randomization integrating GWAS and eQTL data reveals genetic determinants of complex and clinical traits. *Nat. Commun.* 10:3300. 10.1038/s41467-019-10936-0 31341166 PMC6656778

[B47] Ritchie S. J. Tucker-Drob E. M. Cox S. R. Corley J. Dykiert D. Redmond P. (2016). Predictors of ageing-related decline across multiple cognitive functions. *Intelligence* 59 115–126. 10.1016/j.intell.2016.08.007 27932854 PMC5127886

[B48] Salat D. H. Buckner R. L. Snyder A. Z. Greve D. N. Desikan R. S. R. Busa E. (2004). Thinning of the cerebral cortex in aging. *Cereb. Cortex* 14 721–730. 10.1093/cercor/bhh032 15054051

[B49] Savilla K. Kettler L. Galletly C. (2008). Relationships between cognitive deficits, symptoms and quality of life in schizophrenia. *Aust. N. Z. J. Psychiatry* 42 496–504. 10.1080/00048670802050512 18465376

[B50] Sekula P. Del Greco M. F. Pattaro C. Köttgen A. (2016). Mendelian randomization as an approach to assess causality using observational data. *J. Am. Soc. Nephrol.* 27 3253–3265. 10.1681/asn.2016010098 27486138 PMC5084898

[B51] Tyler C. V. Schramm S. C. Karafa M. Tang A. S. Jain A. K. (2011). Chronic disease risks in young adults with autism spectrum disorder: Forewarned is forearmed. *Am. J. Intellect. Dev. Disabil.* 116 371–380. 10.1352/1944-7558-116.5.371 21905805

[B52] Verbanck M. Chen C. Y. Neale B. Do R. (2018). Publisher correction: Detection of widespread horizontal pleiotropy in causal relationships inferred from Mendelian randomization between complex traits and diseases. *Nat. Genet.* 50:1196. 10.1038/s41588-018-0164-2 29967445

[B53] Verdelho A. Madureira S. Ferro J. M. Baezner H. Blahak C. Poggesi A. (2012). Physical activity prevents progression for cognitive impairment and vascular dementia. *Stroke* 43 3331–3335. 10.1161/STROKEAHA.112.661793 23117721

[B54] Vila-Barrios L. Carballeira E. Varela-Sanz A. Iglesias-Soler E. Dopico-Calvo X. (2023). The impact of regular physical exercise on psychopathology, cognition, and quality of life in patients diagnosed with schizophrenia: A scoping review. *Behav. Sci. (Basel)* 13:959. 10.3390/bs13120959 38131815 PMC10740550

[B55] Wassenaar T. M. Wheatley C. M. Beale N. Nichols T. Salvan P. Meaney A. (2021). The effect of a one-year vigorous physical activity intervention on fitness, cognitive performance and mental health in young adolescents: The fit to study cluster randomised controlled trial. *Int. J. Behav. Nutr. Phys. Act* 18:47. 10.1186/s12966-021-01113-y 33789683 PMC8011147

[B56] Weinstein A. A. Ngo D. de Avila L. Price J. K. Golabi P. Austin P. (2023). Association of physical activity and fine motor performance in individuals with type 2 diabetes mellitus and/or non-alcoholic fatty liver disease. *Ann. Med.* 55 1345–1353. 10.1080/07853890.2023.2193422 36974658 PMC10054279

[B57] WHO (2010). *Global recommendations on physical activity for health.* Geneva: WHO. 26180873

[B58] Wichniak A. Skowerska A. Chojnacka-Wójtowicz J. Tafliński T. Wierzbicka A. Jernajczyk W. (2011). Actigraphic monitoring of activity and rest in schizophrenic patients treated with olanzapine or risperidone. *J. Psychiatr. Res.* 45 1381–1386. 10.1016/j.jpsychires.2011.05.009 21679968

[B59] Zimmer P. Baumann F. T. Oberste M. Wright P. Garthe A. Schenk A. (2016). Effects of exercise interventions and physical activity behavior on cancer related cognitive impairments: A systematic review. *Biomed. Res. Int.* 2016:1820954. 10.1155/2016/1820954 27144158 PMC4842032

