# Supplementary material for: Dissecting the causal relationship between moderate to vigorous physical activity levels and cognitive performance: a bidirectional two-sample Mendelian randomization study
Source: Front Psychol. 2024 Sep 6;15:1368241. doi: 10.3389/fpsyg.2024.1368241 (PMC11412864; doi:10.3389/fpsyg.2024.1368241)
Supplement: Supplementary file 1 [file Table_1.DOCX]

Supplementary Material

# Supplementary Figures


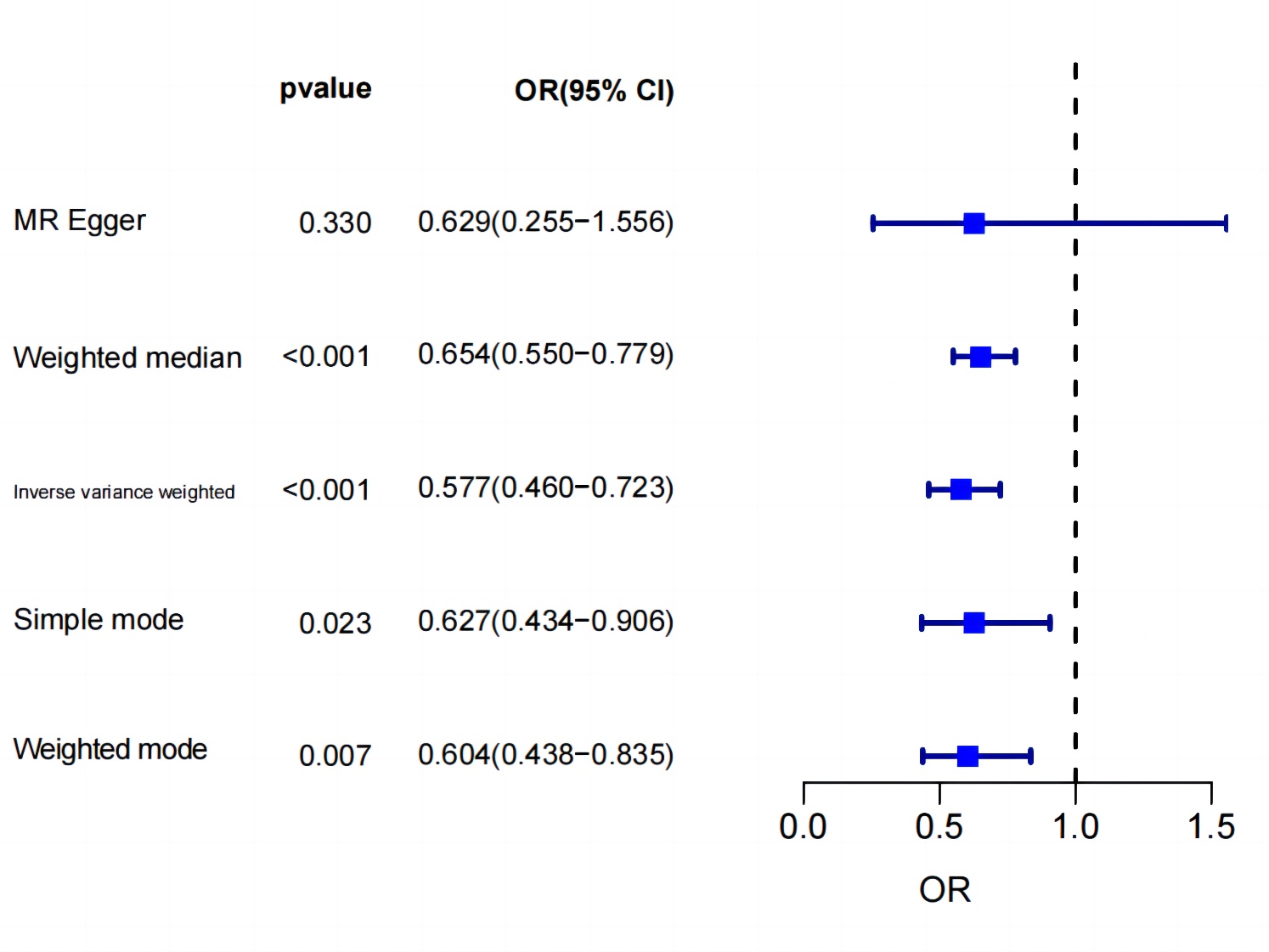


**Supplementary Figure S1**. MR estimation of cognitive performance influenced by MVPA


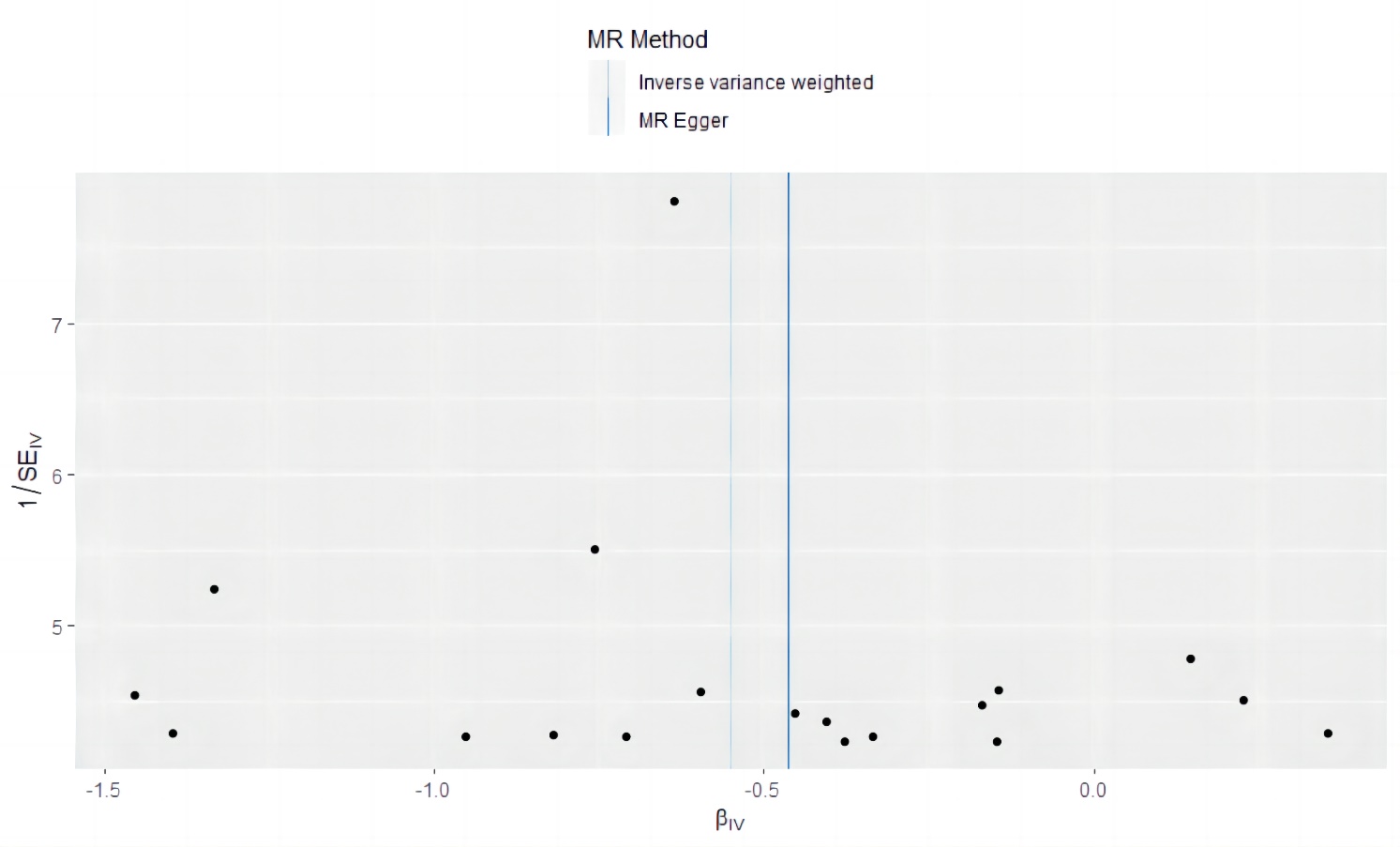


**Supplementary Figure S2**. Funnel diagram depicting the influence of MVPA on cognitive performance


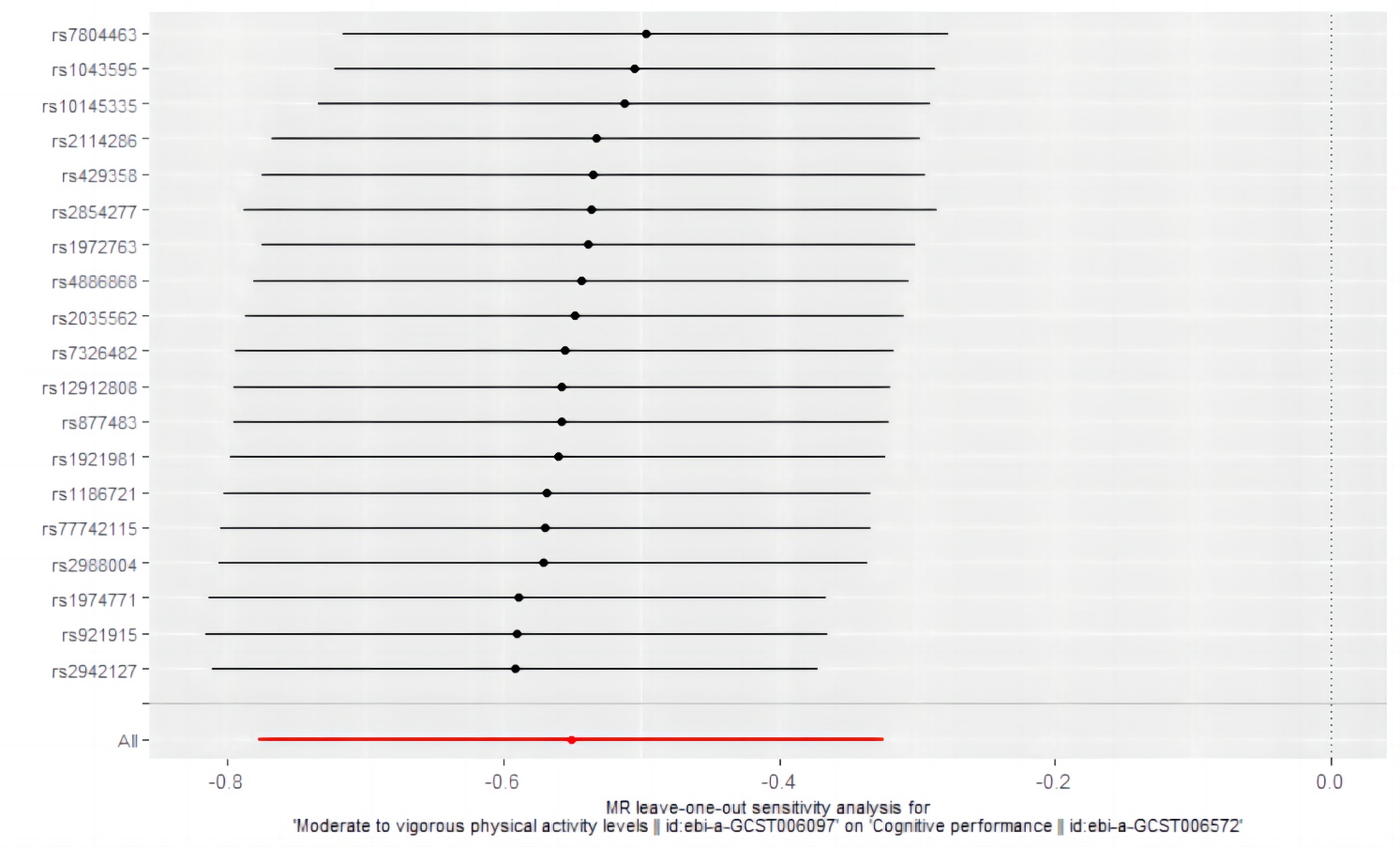


**Supplementary Figure S3**. Analysis of the impact of MVPA on cognitive performance


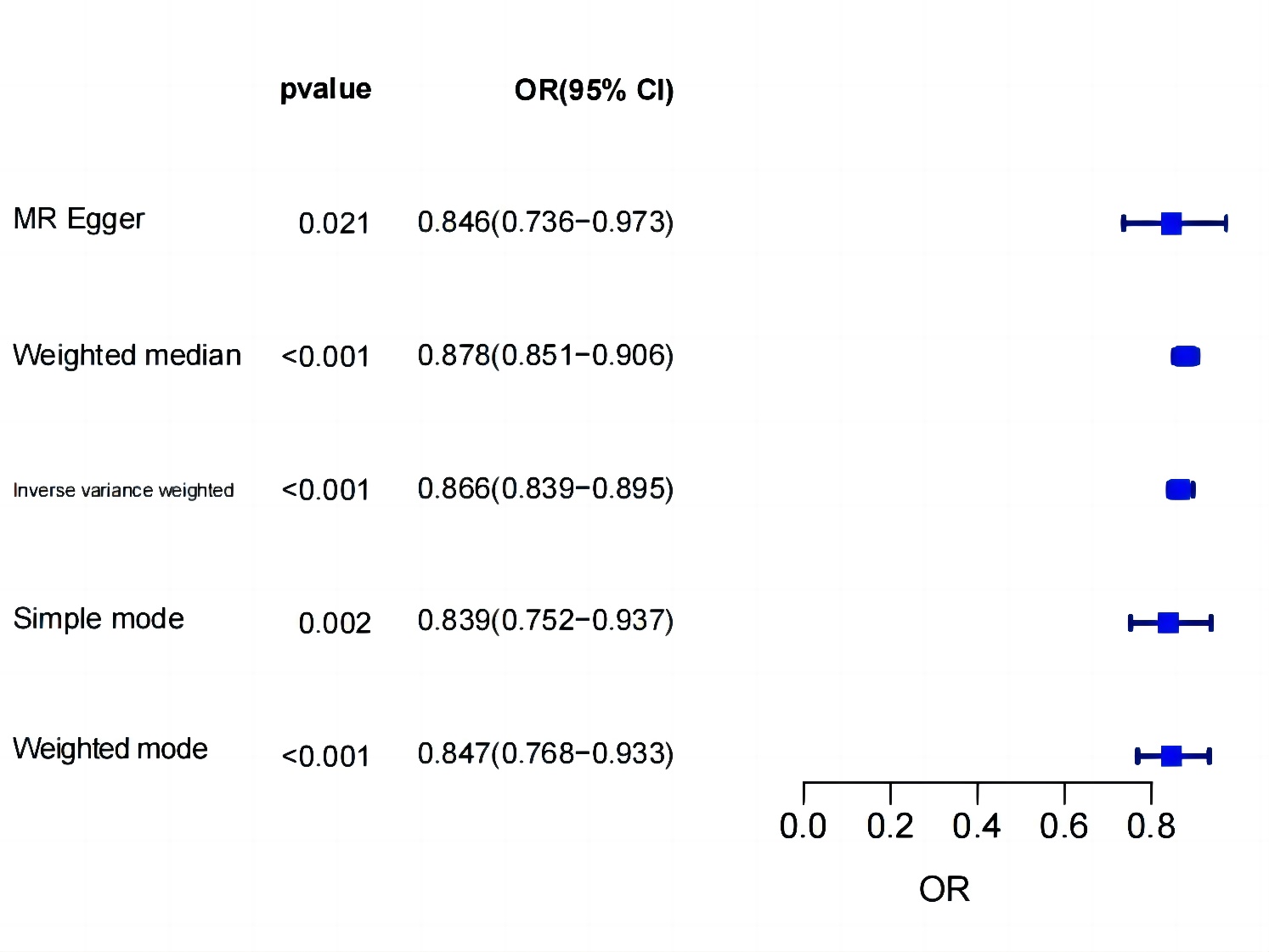


**Supplementary Figure S4**. MR estimation of the impact of cognitive performance on MVPA


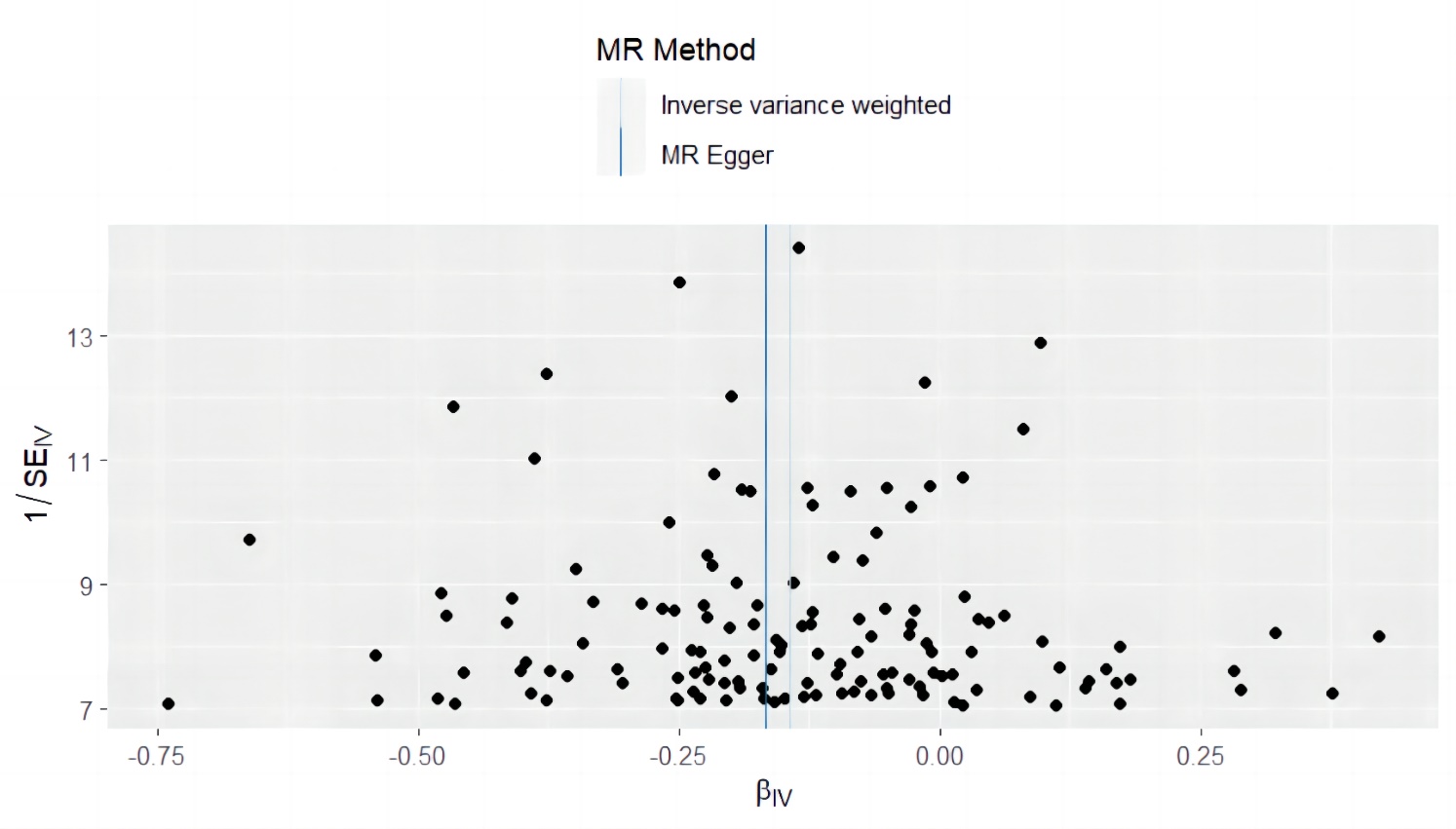
 **Supplementary Figure S5**. Funnel chart illustrating the impact of cognitive performance on MVPA


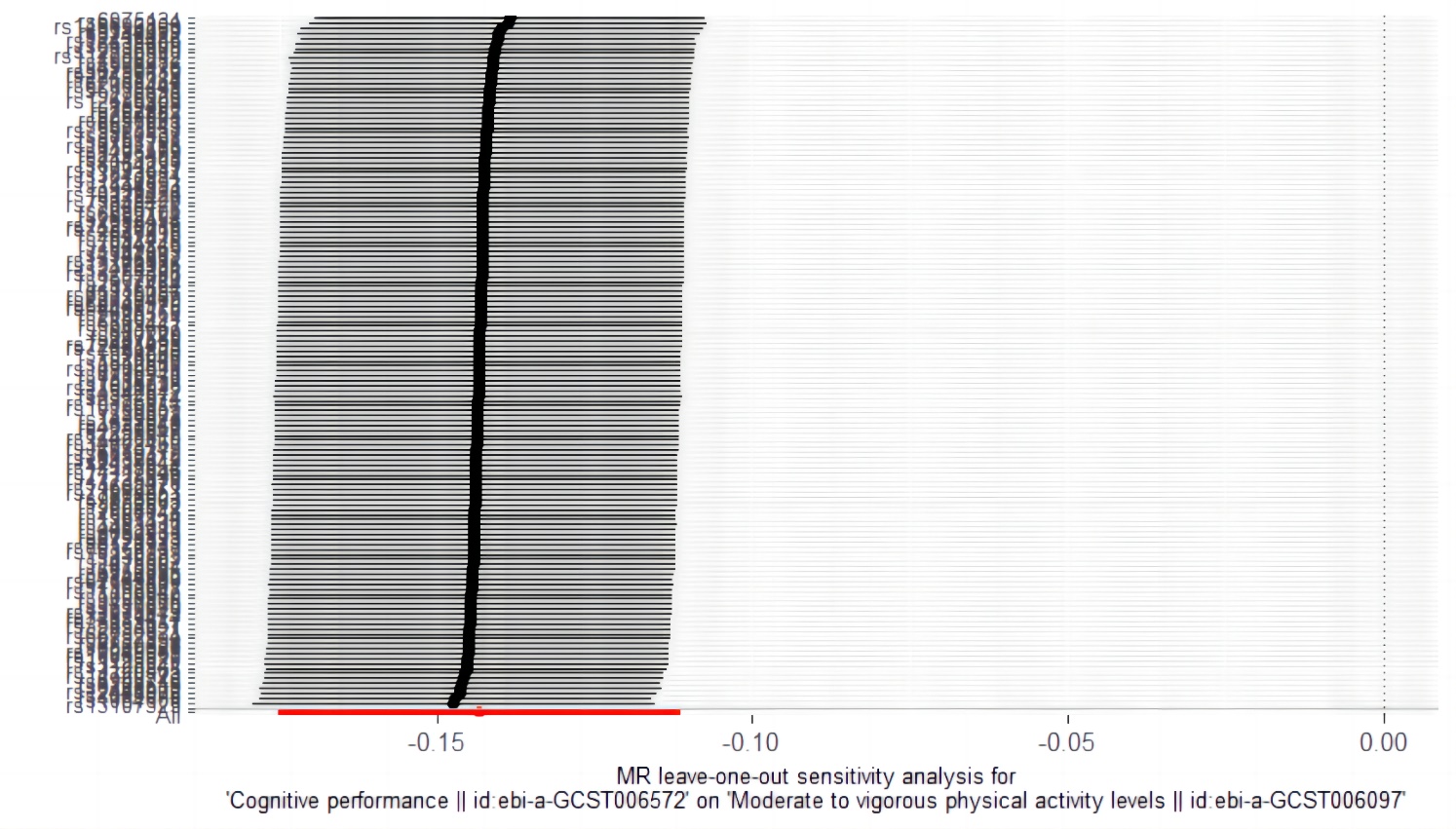
 **Supplementary Figure S6**. Analysis of the effect of cognitive performance on MVPASensitivity analysis
