# Supplementary material for: Dissecting the causal relationship between moderate to vigorous physical activity levels and cognitive performance: a bidirectional two-sample Mendelian randomization study
Source: Front Psychol. 2024 Sep 6;15:1368241. doi: 10.3389/fpsyg.2024.1368241 (PMC11412864; doi:10.3389/fpsyg.2024.1368241)
Supplement: Supplementary file 3 [file Table_3.docx]

**STROBE-MR checklist of recommended items to address in reports of Mendelian randomization studies**^1^ ^2^

| **Item No.** | **Section** | **Checklist item** | **Page No.** | **Relevant text from manuscript** |
| --- | --- | --- | --- | --- |
| 1 | **TITLE and ABSTRACT** | Indicate Mendelian randomization (MR) as the study’s design in the title and/or the abstract if that is a main purpose of the study |  | Dissecting the Causal Relationship Between Moderate to Vigorous Physical Activity Levels and Cognitive Performance: A Bidirectional Two-Sample Mendelian Randomization Study  To address this, we employed a Mendelian randomization (MR) approach, analyzing two distinct samples. |
|  | **INTRODUCTION** |  |  |  |
| 2 | **Background** | Explain the scientific background and rationale for the reported study. What is the exposure? Is a potential causal relationship between exposure and outcome plausible? Justify why MR is a helpful method to address the study question |  | MR is an epidemiological technique that employs single nucleotide polymorphisms (SNPs) associated with an exposure (e.g., MVPA) as instrumental variables (IVs) to deduce potential causal relationships between the exposure and outcomes (Davey Smith & Hemani, 2014). |
| 3 | **Objectives** | State specific objectives clearly, including pre-specified causal hypotheses (if any). State that MR is a method that, under specific assumptions, intends to estimate causal effects |  | This study aims to explore the bidirectional causal effects between MVPA and cognitive performance. |
|  | **METHODS** |  |  |  |
| 4 | **Study design and data sources** | Present key elements of the study design early in the article. Consider including a table listing sources of data for all phases of the study. For each data source contributing to the analysis, describe the following: |  |  |
|  | a) | Setting: Describe the study design and the underlying population, if possible. Describe the setting, locations, and relevant dates, including periods of recruitment, exposure, follow-up, and data collection, when available. |  | We selected major SNPs associated with Moderate To Vigorous Physical Activity (MVPA) and cognitive performance as genetic instrumental variables (IVs) to conduct bidirectional two-sample MR. |
|  | b) | Participants: Give the eligibility criteria, and the sources and methods of selection of participants. Report the sample size, and whether any power or sample size calculations were carried out prior to the main analysis |  | For MVPA, we used publicly available GWAS data (Guthold et al., 2018) from a cohort of 377,234 participants.  The cognitive performance data was sourced from a GWAS (Lee et al., 2018) involving 257,841 participants. |
|  | c) | Describe measurement, quality control and selection of genetic variants |  | Our selection of qualified genetic instruments involved a thorough quality control process. |
|  | d) | For each exposure, outcome, and other relevant variables, describe methods of assessment and diagnostic criteria for diseases |  | Cognitive decline can impair work capacity, social interactions, and daily living activities (Green, 1996). This is particularly concerning when cognitive deficits progress to clinically diagnosed dementia, such as Alzheimer's disease or vascular dementia.  Conversely, insufficient physical activity, as defined by the World Health Organization's guidelines, is a risk factor for non-communicable diseases, including cognitive impairments. These guidelines recommend at least 150 minutes of moderate-intensity or 75 minutes of vigorous-intensity physical activity per week, or a comparable combination of both. |
|  | e) | Provide details of ethics committee approval and participant informed consent, if relevant |  | Our MR analysis of the two samples utilized published Genome-Wide Association Study (GWAS) summary statistics and received ethical approval from the original study. |
| 5 | **Assumptions** | Explicitly state the three core IV assumptions for the main analysis (relevance, independence and exclusion restriction) as well assumptions for any additional or sensitivity analysis |  | Therefore, our research design is outlined as follows: (1) Identification of exposure-related SNPs under a genome-wide significance threshold; (2) Independence of the SNP from potential confounding factors; (3) Influence of the SNP on the outcome exclusively through exposure. |
| 6 | **Statistical methods: main analysis** | Describe statistical methods and statistics used |  |  |
|  | a) | Describe how quantitative variables were handled in the analyses (i.e., scale, units, model) |  | Initially, we included SNPs closely related to exposure factors at a significance threshold of 10-8. We then employed an R2<0.001 and a window size of 10,000 kb for clustering. |
|  | b) | Describe how genetic variants were handled in the analyses and, if applicable, how their weights were selected |  |  |
|  | c) | Describe the MR estimator (e.g. two-stage least squares, Wald ratio) and related statistics. Detail the included covariates and, in case of two-sample MR, whether the same covariate set was used for adjustment in the two samples |  | Finally, we aligned exposure and outcome data to exclude palindromic SNPs and ensure that the effective allele was consistent across both datasets (Hartwig et al., 2016). |
|  | d) | Explain how missing data were addressed |  | In cases where the target SNP was unavailable in the result dataset, a proxy SNP with an R2>0.8 was used as a substitute. |
|  | e) | If applicable, indicate how multiple testing was addressed |  |  |
| 7 | **Assessment of assumptions** | Describe any methods or prior knowledge used to assess the assumptions or justify their validity |  | Our primary method for MR was the IVW regression.  Additionally, we employed complementary methods, including MR Egger regression, the weighted median, the simple model, and the weighted model, to assess the causal impact of exposure on outcomes. |
| 8 | **Sensitivity analyses and additional analyses** | Describe any sensitivity analyses or additional analyses performed (e.g. comparison of effect estimates from different approaches, independent replication, bias analytic techniques, validation of instruments, simulations) |  | We performed a series of sensitivity analyses to evaluate the robustness and validity of our causal estimates. |
| 9 | **Software and pre-registration** |  |  |  |
|  | a) | Name statistical software and package(s), including version and settings used |  | In this study, all analyses were conducted using the TwoSampleMR package (version 0.5.8) and MR-PRESSO (version 1.0) in R software (version 4.3.2). |
|  | b) | State whether the study protocol and details were pre-registered (as well as when and where) |  | The data sources for this study are detailed in Supplementary Table S1. |
|  | **RESULTS** |  |  |  |
| 10 | **Descriptive data** |  |  |  |
|  | a) | Report the numbers of individuals at each stage of included studies and reasons for exclusion. Consider use of a flow diagram |  | These analyses utilized published Genome-wide association study (GWAS) summary statistics for MVPA (n=377,234) and cognitive performance (n=257,841). |
|  | b) | Report summary statistics for phenotypic exposure(s), outcome(s), and other relevant variables (e.g. means, SDs, proportions) |  | Using genetic variables related to MVPA as tools, 19 SNPs were identified as significantly associated with MVPA (P<5 × 10-8) and met the IV criteria.  Here, 147 SNPs were identified as significantly related to cognitive performance (P<5 × 10-8), with 139 of these SNPs meeting the IV criteria. |
|  | c) | If the data sources include meta-analyses of previous studies, provide the assessments of heterogeneity across these studies |  |  |
|  | d) | For two-sample MR:  i.  Provide justification of the similarity of the genetic variant-exposure associations between the exposure and outcome samples  ii.  Provide information on the number of individuals who overlap between the exposure and outcome studies |  | (Supplementary Table S2)  (Supplementary Table S3) |
| 11 | **Main results** |  |  |  |
|  | a) | Report the associations between genetic variant and exposure, and between genetic variant and outcome, preferably on an interpretable scale |  | The MR analysis revealed that MVPA acts as a protective factor against cognitive risk.  Conversely, when cognitive genetic predisposition was considered as the exposure, MR analysis showed a decrease in MVPA deficiency risk with improved cognitive performance |
|  | b) | Report MR estimates of the relationship between exposure and outcome, and the measures of uncertainty from the MR analysis, on an interpretable scale, such as odds ratio or relative risk per SD difference |  | The Causal Effect of MVPA on Cognitive Performance (IVW OR=0.577, 95% CI: 0.460-0.723, p=1.930 × 10-6)  Causality of Cognitive Performance on MVPA (IVW OR=0.866, 95% CI: 0.839-0.895, p=1.200 × 10-18) |
|  | c) | If relevant, consider translating estimates of relative risk into absolute risk for a meaningful time period |  | A logarithmic odds ratio (OR) increase in MVPA correlated with a 42.3% decrease in cognitive risk |
|  | d) | Consider plots to visualize results (e.g. forest plot, scatterplot of associations between genetic variants and outcome versus between genetic variants and exposure) |  | Figure 1 Supplementary Figure S1  Figure 2 Supplementary Figure S4 |
| 12 | **Assessment of assumptions** |  |  |  |
|  | a) | Report the assessment of the validity of the assumptions |  | Cochran's Q statistics were employed to evaluate heterogeneity, which could be attributed to variations in experimental platforms. |
|  | b) | Report any additional statistics (e.g., assessments of heterogeneity across genetic variants, such as *I^2^*, Q statistic or E-value) |  | Table 1  Table 2 |
| 13 | **Sensitivity analyses and additional analyses** |  |  |  |
|  | a) | Report any sensitivity analyses to assess the robustness of the main results to violations of the assumptions |  | The MR Egger test assessed directional pleiotropy (p=0.846), finding no significant directional pleiotropy in all results. |
|  | b) | Report results from other sensitivity analyses or additional analyses |  | The funnel plot also indicated a low risk of directional pleiotropy in our IVW estimates. (Supplementary Figure S2)  (Supplementary Figure S5) |
|  | c) | Report any assessment of direction of causal relationship (e.g., bidirectional MR) |  | We discovered that MVPA can mitigate cognitive risk, yet a low cognitive level also contributes to inadequate MVPA. |
|  | d) | When relevant, report and compare with estimates from non-MR analyses |  |  |
|  | e) | Consider additional plots to visualize results (e.g., leave-one-out analyses) |  | The stability of the results was confirmed through single SNP exclusion analysis. (Supplementary Figure S3) (Supplementary Figure S6) |
|  | **DISCUSSION** |  |  |  |
| 14 | **Key results** | Summarize key results with reference to study objectives |  | In our MR study, a significant potential causal relationship between MVPA and cognitive performance was identified. We observed that MVPA has a protective effect. |
| 15 | **Limitations** | Discuss limitations of the study, taking into account the validity of the IV assumptions, other sources of potential bias, and imprecision. Discuss both direction and magnitude of any potential bias and any efforts to address them |  | However, it is important to acknowledge certain limitations.  Additionally, due to the limited information in the aggregated data, we are unable to ascertain if gender influences our results. |
| 16 | **Interpretation** |  |  |  |
|  | a) | Meaning: Give a cautious overall interpretation of results in the context of their limitations and in comparison with other studies |  |  |
|  | b) | Mechanism: Discuss underlying biological mechanisms that could drive a potential causal relationship between the investigated exposure and the outcome, and whether the gene-environment equivalence assumption is reasonable. Use causal language carefully, clarifying that IV estimates may provide causal effects only under certain assumptions |  |  |
|  | c) | Clinical relevance: Discuss whether the results have clinical or public policy relevance, and to what extent they inform effect sizes of possible interventions |  | From an individual perspective, we strongly advocate for all adults to engage in at least 150 minutes per week of MVPA to maintain brain health and lessen the risk of age-related cognitive disorders.  On a national level, we recommend the implementation of effective policies to encourage non-motorized commuting, such as walking and cycling, by enhancing infrastructure and road safety. |
| 17 | **Generalizability** | Discuss the generalizability of the study results (a) to other populations, (b) across other exposure periods/timings, and (c) across other levels of exposure |  | The GWAS summary data utilized in this study encompasses only individuals of European descent. Consequently, the applicability of our findings to other populations requires further validation. |
|  | **OTHER INFORMATION** |  |  |  |
| 18 | **Funding** | Describe sources of funding and the role of funders in the present study and, if applicable, sources of funding for the databases and original study or studies on which the present study is based |  | Ministry of Education's Collaborative Education Initiative: An Exploration of Teaching Models for AR-Enhanced Aerobic Fitness Courses (Project Number: 202002064006) |
| 19 | **Data and data sharing** | Provide the data used to perform all analyses or report where and how the data can be accessed, and reference these sources in the article. Provide the statistical code needed to reproduce the results in the article, or report whether the code is publicly accessible and if so, where |  | The original data and code files are stored in the Open Science Framework (https://osf.io/bhp4c/). |
| 20 | **Conflicts of Interest** | All authors should declare all potential conflicts of interest |  | The authors declare that the research was conducted in the absence of any commercial or financial relationships that could be construed as a potential conflict of interest. |

This checklist is copyrighted by the Equator Network under the Creative Commons Attribution 3.0 Unported (CC BY 3.0) license.

1. **Skrivankova, V.W., Richmond, R.C., Woolf, B.A.R., Davies, N.M., Swanson, S.A., VanderWeele, T.J., Timpson, N.J., Higgins, J.P.T., Dimou, N., Langenberg, C., Loder, E.W., Golub, R.M., Egger, M., Davey Smith, G., and Richards, J.B. (2021a).** Strengthening the reporting of observational studies in epidemiology using mendelian randomisation (STROBE-MR): Explanation and elaboration. *BMJ* **375**,n2233. doi:10.1136/bmj.n2233

2. **Skrivankova, V.W., Richmond, R.C., Woolf, B.A.R., Yarmolinsky, J., Davies, N.M., Swanson, S.A., VanderWeele, T.J., Higgins, J.P.T., Timpson, N.J., Dimou, N., Langenberg, C., Golub, R.M., Loder, E.W., Gallo, V., Tybjaerg-Hansen, A., Davey Smith, G., Egger, M., and Richards, J.B. (2021b).** Strengthening the Reporting of Observational Studies in Epidemiology Using Mendelian Randomization: The STROBE-MR Statement. *JAMA* **326**,1614-1621. doi:10.1001/jama.2021.18236
